# Synergistic enhancement of PARP inhibition via small molecule UNI66-mediated suppression of BRD4-dependent transcription of *RAD51* and *CtIP*

**DOI:** 10.1093/narcan/zcaf013

**Published:** 2025-04-30

**Authors:** Enkhzul Amarsanaa, Minwoo Wie, Unbeom Shin, Nabeela Bilal, Jungme Hwang, Eun A Lee, Seon Young Lee, Byung-Gyu Kim, Shinseog Kim, Yoonsung Lee, Kyungjae Myung

**Affiliations:** Center for Genomic Integrity, Institute for Basic Science, Ulsan 44919, Republic of Korea; Department of Biological Sciences, Ulsan National Institute of Science and Technology, Ulsan 44919, Republic of Korea; Center for Genomic Integrity, Institute for Basic Science, Ulsan 44919, Republic of Korea; Department of Biological Sciences, Ulsan National Institute of Science and Technology, Ulsan 44919, Republic of Korea; Center for Genomic Integrity, Institute for Basic Science, Ulsan 44919, Republic of Korea; Center for Genomic Integrity, Institute for Basic Science, Ulsan 44919, Republic of Korea; Department of Biomedical Engineering, Ulsan National Institute of Science and Technology, Ulsan 44919, Republic of Korea; Center for Genomic Integrity, Institute for Basic Science, Ulsan 44919, Republic of Korea; Center for Genomic Integrity, Institute for Basic Science, Ulsan 44919, Republic of Korea; Center for Genomic Integrity, Institute for Basic Science, Ulsan 44919, Republic of Korea; Center for Genomic Integrity, Institute for Basic Science, Ulsan 44919, Republic of Korea; Center for Genomic Integrity, Institute for Basic Science, Ulsan 44919, Republic of Korea; Clinical Research Institute, Kyung Hee University Hospital at Gangdong, School of Medicine, Kyung Hee University, Seoul 05278, Republic of Korea; Center for Genomic Integrity, Institute for Basic Science, Ulsan 44919, Republic of Korea; Department of Biomedical Engineering, Ulsan National Institute of Science and Technology, Ulsan 44919, Republic of Korea

## Abstract

Targeted therapy leveraging synthetic lethality in homologous recombination (HR)-defective tumors, particularly in BRCA-mutated tumors through poly(ADP-ribose) polymerase (PARP)-dependent repair inhibition, has shown success. However, the challenge lies in the ability of the tumors to reactivate HR via diverse mechanisms, leading to resistance against PARP-dependent repair inhibition. Addressing this issue, the down-regulation of HR activity has been explored as a potential strategy to overcome PARP inhibitor-resistant tumors. Yet, the intricate modulation of HR gene expression in mammalian cells is still not fully understood. In this study, we used a small molecule, UNI66, identified from high-throughput screening, to investigate regulatory mechanisms of HR. UNI66 was observed to induce synthetic lethality in PARP1-deficient cells and enhanced the sensitivity of multiple cancer cells to PARP inhibitors, suggesting a role in HR down-regulation. Mechanistically, UNI66 was found to interact with and inhibit BRD4 protein binding to the promoters of *CtIP* and *RAD51* genes, resulting in the down-regulation of their transcription. This decrease in *CtIP* and *RAD51* expression was associated with reduced HR activity, thereby increasing the sensitivity of tumors to PARP inhibitors. These findings indicate that BRD4-mediated transcriptional regulation of *CtIP* and *RAD51* influences HR activity, which may have implications for overcoming resistance to PARP inhibitors.

## Graphical Abstract

**Graphical Abstract:**
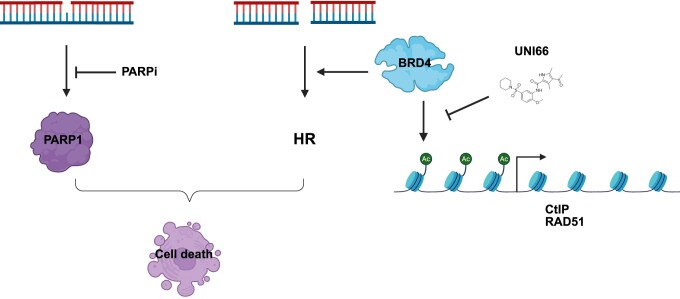

## Introduction

Cancer cells frequently lose one or more DNA repair pathways, leading to genomic instability and an increased mutation burden [1]. The loss of a specific DNA repair pathway makes cancer cells heavily reliant on alternative repair mechanisms for survival during continuous cell division. Exploiting this dependency on target-specific repair pathways presents an opportunity for a precision therapeutic approach through synthetic lethality [2, 3]. Synthetic lethality, a phenomenon where the deletion of one gene is viable but the simultaneous deletion of two or more genes results in cell death due to their redundant functions for survival, has proven to be a successful strategy in cancer therapy [4]. Notably, poly(ADP-ribose) polymerase (PARP) inhibitors have emerged as a prominent example. PARP inhibitors are employed in the treatment of cancer cells with mutations in *BRCA1* or *BRCA2*, which are deficient in homologous recombinational (HR) repair for repairing DNA double-strand breaks (DSBs). By inhibiting PARP activity, the repair or response to single-strand breaks (SSBs) or gaps in DNA is compromised, leading to the generation of DNA DSBs after DNA replication. The accumulation of these DNA DSBs results in cell death specifically in DNA DSB repair-deficient cancer cells carrying *BRCA1* or *BRCA2* mutations through synthetic lethality [5, 6]. Moreover, PARP inhibition induces synthetic lethality in *BRCA1* or *BRCA2* mutated tumors due to the redundant roles of PARPs in protecting stalled replication forks and repairing DNA gaps [7, 8]. This multifaceted approach exploits the dependency of cancer cells on alternative repair pathways, offering a targeted and effective strategy for treating specific types of DNA repair-deficient cancers.

Beyond the well-established synthetic lethal relationship between PARP1 and BRCA pairs, numerous other potential synthetic lethal pairs have been unveiled, holding significant promise for clinical applications. One illustrative example involves the inhibition of DNA polymerase θ (POLθ), a key player in the alternative DNA DSB repair pathway known as POLθ-mediated end joining (TMEJ), which demonstrates synthetic lethality with *BRCA1* or *BRCA2* mutations [9, 10]. Another intriguing instance is the inhibition of WRN, a helicase pivotal in various DNA repair processes, resulting in synthetic lethality with mismatch repair deficiency commonly observed in microsatellite instability (MSI) tumors [11]. Notably, synthetic lethal pairs extend beyond DNA repair pathways and into other cellular processes. For instance, the combination of a p53 activator (RG) and a Bcl2 inhibitor (ABT) has demonstrated synthetic lethality. Given that Bcl2 and p53 regulate apoptotic cell death by suppression and activation, respectively, the inhibition of Bcl2 by ABT induces apoptosis in cancer cells arrested in the G_1_ phase. This effect is further intensified by p53 activation through RG [12]. This innovative synthetic lethal approach, encompassing various cellular pathways, emerges as a promising avenue for precision medicine in cancer targeting.

Utilizing small molecules as tools offers a powerful approach to uncover novel synthetic lethal pairs, deepening our understanding of DNA repair pathways and opening up new avenues for targeted drug discovery. By selectively perturbing specific genes or enzymes involved in these pathways, researchers have identified genetic interactions where the disruption of one pathway, combined with other genetic deficiencies, induces cell death—thereby revealing synthetic lethal pairs [13]. We previously identified 289 small molecules capable of enhancing DNA replication stresses from the NIH Molecular Library Probe Production Centers Network (MLPCN) library comprising 344 385 small molecules. To conduct this screening, we employed a HEK293T cell line engineered to express the luciferase-tagged *ATAD5* gene, utilizing luciferase expression as an informative signal for detecting DNA replication stress [14]. ATAD5, or ATPase family AAA domain-containing protein 5, serves as a valuable biomarker for identifying compounds inducing DNA replication stress due to its characteristic elevation following such stress [15]. ATAD5 plays a critical role in ensuring DNA replication and preserving genomic integrity. It regulates the dynamics of proliferating cell nuclear antigen (PCNA) during DNA replication and repair by unloading PCNA from DNA post-DNA synthesis through its ATPase activity. Additionally, ATAD5 facilitates the deubiquitination of PCNA after DNA damage bypass through its interaction with the deubiquitinating enzyme for PCNA, USP1-UAF1 [16, 17]. Employing a synthetic lethal approach, we characterized the efficacy of a specific small molecule, baicalein, in selectively eliminating mismatch repair-deficient tumors. Baicalein achieves this selectivity through its preferential interaction with mismatched DNA and the MSH2–MSH6 complex, ultimately activating the ATM–CHK2 pathway [18]. Furthermore, our investigations led to the discovery of another intriguing small molecule, 2-chloro-*N*,*N*-diethylethanamine hydrochloride (CDEAH), which induces the formation of DNA adducts requiring both PARP1-dependent DNA repair and nucleotide excision repair pathways for resolution [19]. These findings highlight the potential of using small molecules as tools to deepen our understanding of DNA replication and repair processes, providing valuable insights for potential therapeutic interventions in cancer treatment.

BRD4, a member of the bromodomain and extra-terminal domain (BET) family proteins, plays a pivotal role in cellular processes through its distinct structure comprising extra-terminal domains and two N-terminal bromodomains (BD1 and BD2). Functionally, BRD4 recognizes and binds to acetylated lysine residues on histones using its bromodomains [20]. This recognition allows BRD4 to accumulate at specific gene promoters and enhancers, where it recruits the positive transcription elongation factor b (P-TEFb), thereby promoting RNA polymerase II transcription initiation and elongation [21]. Recent studies have expanded our understanding of BET family proteins, revealing their direct involvement in DNA damage repair pathways and their roles at DNA replication forks for proper PCNA cycling [22–24]. Notably, BRD4 inhibition has been shown to reduce the transcription of *CtIP*, resulting in decreased end resection and a defect in HR repair. This defect makes cells more sensitive to PARP inhibitors, positioning BRD4 as a potential target for combinational therapy [25, 26]. Recent advancements have led to the development of novel inhibitors specifically targeting BET proteins, offering exciting prospects for therapeutic interventions [27, 28]. For instance, the BRD4-specific inhibitor JQ1 effectively displaces BRD4 from chromatin, particularly from the *CtIP* promoter and enhancer regions. Consequently, JQ1 treatment significantly reduces HR activity. Combinational therapy involving JQ1 and PARP inhibitors has been proposed as a promising strategy for treating various tumors, including those resistant to PARP inhibitors [29, 30]. These insights into the multifaceted roles of BRD4 and the therapeutic potential of BET inhibitors underscore the ongoing progress in developing targeted and effective strategies for cancer treatment.

In our current research, we conducted a comprehensive screening of previously identified small molecules known to enhance DNA replication stress in order to investigate their potential for synthetic lethality with PARP1 deficiency. In addition to the previously identified compound CDEAH, our study unveiled another promising candidate, 4-acetyl-*N*-(2-methoxy-5-piperidin-1-ylsulfonylphenyl)-3,5-dimethyl-1H-pyrrole-2-carboxamide, referred to as UNI66, which exhibited selective lethality towards PARP1-deficient cells. Upon further investigation and characterization of UNI66, we identified its capacity to inhibit the BRD4 protein. This inhibition of BRD4 by UNI66 had significant downstream effects, specifically in the suppression of transcription of the *CtIP* and *RAD51* genes. The consequence of this inhibition was the induction of defects in HR. Our study unveils UNI66 as a novel BRD4 inhibitor to suppress transcriptional suppression of the HR genes including *CtIP* and *RAD51*. These findings not only contribute to our understanding of the intricate molecular interactions underlying DNA replication stress and repair mechanisms but also propose a novel therapeutic avenue for targeted and synergistic treatments of BRD4 inhibition, particularly in the context of PARP inhibitor-resistant tumors.

## Materials and methods

### Cell lines

HCT116 (ATCC), U2OS (ATCC), Capan, C2-2, and HEK293T ATAD5-LUC [14] cells were cultured in Dulbecco’s modified Eagle’s medium (DMEM) (Gibco), while HAP1 (Horizon) cells were cultured in Iscove’s modified Dulbecco's medium (IMDM). The culture medium for all cell lines consisted of 10% fetal bovine serum (FBS) (Merck) and 1% penicillin and streptomycin (Invitrogen). U2OS cells stably expressing DR–green fluorescent protein (GFP) and EJ5–GFP were cultured in DMEM (Gibco) supplemented with 10% FBS (Merk) and 2 μg/ml puromycin (Invitrogen). TK6 BRCA2-degron cells were maintained in RPMI-1640 medium containing 5% horse serum (Gibco), 10 mg/ml sodium pyruvate (Gibco), and 1% penicillin and streptomycin (Invitrogen). Cultures were maintained at 37°C in a 5% CO_2_ atmosphere.

### Chemicals

The chemical compound 4-acetyl-*N*-(2-methoxy-5-piperidin-1-ylsulfonylphenyl)-3,5-dimethyl-1H-pyrrole-2-carboxamide (UNI66; catalog number EN300-26923464) was obtained from Enamine. JQ1 (catalog number SML1524) and MG132 (Z-Leu-Leu-Leu-al, catalog number C2211-5MG) were acquired from Sigma-Aldrich. Olaparib (AZD2281) was obtained from Selleckchem, and Mirin (catalog number 475954-10MG) was sourced from Merck Millipore. POLQ inhibitor ART558 (catalog number HY-141520) was purchased from MedChemExpress.

### Antibodies

The following antibodies were purchased from the specified suppliers: anti-CtIP (9201S) and anti-RAD51 (8875) were obtained from Cell Signaling Technology. Anti-BRD4 (A301-985A50), anti-BRD3 (A302-368A), and anti-BRD2 (A302-583A) were purchased from Bethyl Laboratories. Anti-α-tubulin (DM1A, sc-32293) was acquired from Santacruz. Anti-γ-H2AX (Ser139, 05–636), anti-RPA2 (MABE285), and anti-Histone H3 (07-690) antibodies were purchased from Merck Millipore. Anti-PARP1 (ALX-210-302-R100) was obtained from Enzo Life Sciences.

### ATAD5–luciferase assay

HEK293T cells, engineered to stably express ATAD5–luciferase, were seeded at a density of 15,000 cells per well in a 96-well flat-bottom white plate (Costar). Following a 24 h incubation period, the cells were treated with either 5-fluorodeoxyuridine (5-FUrd) or the specified compounds. After an additional 24 h, luciferase activity was assessed by adding One-Glo luciferase reagent (Promega) to the plates. Luminescence intensity was then measured using a Synergy NEO2 Hybrid Multi-Mode Reader (BioTek), following the manufacturer’s protocol.

### Immunoblot analysis

Whole-cell extracts were obtained by incubating cells with RIPA buffer (50 mM Tris–HCl, pH 8.0, 150 mM NaCl, 5 mM EDTA, 1% Triton X-100, 0.1% sodium dodecyl sulfate (SDS), 0.5% sodium deoxycholate, and Halt™ Protease & Phosphatase Single-Use Inhibitor Cocktail) containing Benzonase nuclease (250 U/μl, Enzynomics) on ice for 1 h. The mixture was then sonicated and centrifuged. Proteins were separated by SDS–polyacrylamide gel electrophoresis (PAGE) and transferred onto a nitrocellulose membrane. The membrane underwent a 1 h incubation in Tris-buffered saline (TBS) containing 0.1% Tween-20 (TBS-T) supplemented with 5% skim milk for blocking, followed by an overnight incubation with a primary antibody at 4°C. After washing, the blots were incubated with a horseradish peroxidase-conjugated secondary antibody (Enzo Life Sciences) at a 1:10,000 dilution in TBS-T for 1 h at room temperature. Signal detection was performed using an enhanced chemiluminescent reagent (Thermo Fisher Scientific) and captured by an automated imaging system (ChemiDoc™; Bio-Rad Laboratories), following the manufacturer’s protocol.

### Chromatin fractionation

Cell lysis was performed using buffer A (100 mM NaCl, 300 mM sucrose, 3 mM MgCl_2_, 10 mM PIPES, pH 6.8, 1 mM EGTA, 0.2% Triton X-100, and Halt™ Protease & Phosphatase Single-Use Inhibitor Cocktail) on ice for 10 min. The lysates were then centrifuged at 5000 *g* at 4°C for 5 min to separate the chromatin-containing pellet. The resulting supernatants were collected as the soluble fraction. The pellet, containing chromatin, was subjected to digestion with 50 U of Benzonase (Enzynomics) for 1 h in RIPA buffer [50 mM Tris–HCl, pH 8.0, 150 mM NaCl, 5 mM EDTA, 1% Triton X-100, 0.1% SDS, 0.5% Na deoxycholate, 1 mM phenylmethylsulfonyl fluoride (PMSF), 5 mM MgCl_2_, and Halt™ Protease & Phosphatase Single-Use Inhibitor Cocktail) to extract chromatin-bound proteins. The chromatin-containing fractions were clarified by centrifugation at 15,000 rpm at 4°C for 10 min to remove debris. The protein concentration in each fraction was determined using the Bradford assay (Bio-Rad), and the extracted proteins were subsequently analyzed by immunoblotting.

### Cell cycle analysis

Cells were harvested following trypsinization, washed with phosphate-buffered saline (PBS), and fixed overnight with 70% ethanol in PBS. Subsequently, fixed cells were washed with PBS and treated with 0.2 mg/ml RNase A in PBS at 37°C for 1 h in the dark. DNA was stained with 10 μg/ml propidium iodide (PI) in PBS. Flow cytometry analysis (FACS) was conducted using a FACSVerse™ flow cytometer equipped with BD FACSuite™ software (BD Biosciences). The acquired data were analyzed using the FlowJo software.

### Cell viability

A total of 5,000 HCT116 or HAP1 cells were seeded in a 96-well flat-bottom white plate (Costar). Following a 24 h incubation period, cells were treated with the specified compounds and incubated for 3 days. Cell viability was assessed using Cell Titer-Glo (Promega) according to the manufacturer’s protocol. The assay involves measuring luminescence to quantify cellular ATP levels, providing an indication of cell viability.

### Double-strand break reporter assay

A total of 1 × 10^5^ U2OS cells stably expressing DR–GFP or EJ5–GFP reporter were plated in a 12-well plate. The cells were co-transfected with 0.5 μg of either I-SceI expression vector or an empty vector along with 0.1 μg of a dsRED vector (utilized as a transfection control). The transfections were carried out using 0.1 ml of Opti-mem containing Lipofectamine 3000 (Invitrogen). Subsequently, the percentage of GFP-positive cells was analyzed using the Becton Dickinson FACS Verse flow cytometer (BD Biosciences, San Jose, CA, USA). This flow cytometry-based analysis helps assess the efficiency of DNA repair via quantifying the percentage of cells expressing GFP.

### DNA end resection assay

The protocol for measuring the level of resection adjacent to specific DSBs in U2OS cells stably expressing ER-AsiSI is as follows. U2OS cells stably expressing ER-AsiSI were trypsinized, harvested, and resuspended in PBS (Lonza, Basel, Switzerland) containing 0.6% low-gelling point agarose (Bio-Rad, Hercules, CA, USA) at 37°C. Then, 50 μl of the cell suspension was dropped on a piece of parafilm to create a solidified agar ball, which was then transferred to a 1.5 ml tube. The agar ball was treated with 1 ml of ESP buffer (0.5 M EDTA, 2% *N*-lauroylsarcosine, 1 mg/ml proteinase K, and 1 mM CaCl_2_, pH 8.0) for 20 h at 16°C with rotation, followed by 1 ml of HS buffer (1.85 M NaCl, 0.15 M KCl, 5 mM MgCl_2_, 2 mM EDTA, 4 mM Tris, and 0.5% Triton X-100, pH 7.5) for 20 h at 16°C with rotation. After six washes with 1 ml of phosphate buffer (8 mM Na_2_HPO_4_, 1.5 mM KH_2_PO_4_, 133 mM KCl, and 0.8 mM MgCl_2_, pH 7.4) for 1 h each time at 4°C with rotation, the agar ball was melted by placing the tube in a 70°C heat block for 10 min. The melted sample was diluted 15-fold with 70°C distilled water, mixed with 10× NEB restriction enzyme buffer, and stored at 4°C. For the measurement of resection adjacent to specific DSBs, 20 μl of genomic DNA was digested with 20 U of restriction enzymes (BsrGI and HindIII-HF; New England Biolabs, Ipswich, MA, USA) or mock digested at 37°C overnight. Then, 3 μl of digested or mock-digested samples was used as template in a quantitative polymerase chain reaction (qPCR) with a total volume of 25 μl containing 12.5 μl of 2x TaqMan Universal PCR Master Mix (Thermo Fisher), 0.5 mM of each primer, and 0.2 mM of the probe using a ViiATM 7 Real-Time PCR System (Thermo Fisher). The percentage of single-stranded DNA (ssDNA%) generated by resection was determined with the following equation: ssDNA% = 1/[2(ΔCt − 1) + 0.5] × 100. The percentage of ssDNA in each sample was analyzed using Prism8 software (Version 8.01, GraphPad).

### Apoptosis assay

Apoptotic cell analysis was performed by staining cells with Annexin V, which binds to phosphatidylserine exposed on the outer membrane of apoptotic cells, and a vital dye, such as PI, to differentiate between early and late stages of apoptosis. Flow cytometry is then employed to analyze the fluorescence signals, providing information on the extent of apoptotic cell populations. Briefly, HCT116 wild-type (WT) and HCT116 *parp1*-deficient cells were seeded in 60 mm dishes. The following day, cells were treated with 20 μM UNI66. After 3 days, cells were harvested by trypsinization. Subsequently, cells were incubated in staining buffer containing Annexin V–Alexa Fluor™ 488 (A13201, Thermo Fisher Scientific) and PI at room temperature for 15 min. Flow cytometry was then performed to analyze the fluorescence signals, providing information on the extent of apoptotic cell populations. The acquired data were analyzed using FlowJo software.

### Colony-forming assay

HCT116 WT or PARP1-deficient cells were seeded in 6-well plates at a density of 500 or 700, respectively. Cells were treated with the indicated concentrations of UNI66 and incubated for 10 days. Cells were stained with methylene blue and counted using the Image J program.

### RNA interference

Small interfering RNAs (siRNAs) were purchased from Bioneer and were transfected using RNAiMAX according to the manufacturer's protocol.

### Quantitative real-time PCR

The procedure for gene expression analysis involved RNA extraction, cDNA synthesis, and qPCR, with normalization to a reference gene for accurate and meaningful results. Briefly, total RNA was extracted using Qiazol Reagent (Qiagen) following the manufacturer’s protocol. A 1 μg aliquot of RNA was utilized for cDNA synthesis using the SuperScript IV First-Strand Synthesis System (Invitrogen). This system is designed for high-performance cDNA synthesis, ensuring reliable and accurate representation of mRNA in the cDNA pool. Reverse transcription–qPCR was performed using SYBR Green Master Mix (Applied Biosystems) on a QuantStudio 7 Flex system (Applied Biosystems), following the manufacturer’s protocol. Gene expression levels were normalized to the expression of the housekeeping gene RPLP0. This step is crucial to correct for variations in RNA input and reverse transcription efficiency, providing a reliable measure of the target gene’s expression relative to an internal control.

The following primers were used:

- *RAD51*-forward (5′-TCTCTGGCAGTGATGTCCTGGA-3′);
- *RAD51*-reverse (5′-TAAAGGGCGGTGGCACTGTCTA-3′);
- *CtIP* forward (5′-CAGGAACGAATCTTAGATGCACA-3′);
- *CtIP* reverse (5′-GCCTGCTCTTAACCGATCTTCT-3′);
- *WRN*-forward (5′-CTTTATTCCAAGAAAAGAAGATGCCTTTG-3′);
- *WRN*-reverse (5′-CCTGCTCGCTCCAAATCAAG-3′);
- *RPLP0*-forward (5′-AGCCCAGAACACTGGTCTC-3′);
- and *RPLP0*-reverse (5′-ACTCAGGATTTCAATGGTGCC-3′).

### Promoter assay

In a 6-well plate, 1.5 × 10^5^ HCT116 cells were initially seeded and subsequently co-transfected with a luciferase-expressing plasmid harboring the *RAD51* promoter region along with an mCherry plasmid. Two days post-transfection, the cells underwent treatment with 20 μM UNI66 for 18 h. Analysis was conducted using the dual luciferase reporter assay system following the manufacturer’s protocol. Transfection efficiency was quantified by assessing the population of cells expressing mCherry through flow cytometry.

### Chromatin immunoprecipitation

Chromatin immunoprecipitation (ChIP) was executed utilizing the SimpleChIP Enzymatic Chromatin IP Kit, following the manufacturer’s instructions. In brief, 8 × 10^6^ HCT116 cells were seeded in a 150 mm dish. Subsequently, after a 24 h incubation, the cells were treated with 60 μM UNI66 for 6 h, cross-linked with formaldehyde, and then harvested. Cell lysis was carried out using buffer A, and nuclear fractions were subjected to digestion with micrococcal nuclease, followed by sonication using Covaris S220. A total of 5 μg of chromatin was then incubated with the respective antibody overnight at 4°C with gentle rotation. The antibody–chromatin complexes were isolated utilizing magnetic protein A/G beads, followed by a series of washes. DNA was eluted, de-cross-linked with proteinase K, purified, and subsequently subjected to qPCR.

The following primers were used:

- *RAD51*-promoter-forward, 5′-GAGCTCCCGTCTTGGGTTAG-3′;
- *RAD51*-promoter-reverse, 5′-CGTCGACGTAACGTATCCCC-3′;
- *RAD51*-gene body-forward, 5′-CACCAAAGGCGGTCAGAGAT-3′;
- *RAD51*-gene body-reverse, 5′-ACCTGGAAGCTTTCCTAACTAGAG-3′;
- *CtIP*-P1-forward, 5′-ATTGTCGTCGTGCCTCGAAT-3′;
- *CtIP*-P1-reverse, 5′-AAACCCTTTCCACCTACCCG-3′;
- *CtIP*-P2-forward, 5′-CACCCAGGCAAATGTTTGGTC-3′;
- *CtIP*-P2-reverse, 5′-GCTTAGCCTTGAGGAGCGAG-3′;
- *CtIP*-E3-forward, 5′-ATTATGTCGCCGGAACTGGT-3′;
- *CtIP*-E3-reverse, 5′-AGACCGAGGAAGACCTGACT-3′;
- *Myc*-P-forward, 5′-ACACTAACATCCCACGCTCTG-3′;
- *Myc-*P-reverse, 5′-GATCAAGAGTCCCAGGGAGAGTG-3′;
- *WRN-*P-forward, 5′-CTCCGCTCTTCCTCAGTGC-3′;
- and *WRN*-P-reverse, 5′-ATCCGGAGAAGTGAGAACATTCCC-3′

### Cellular thermal shift assay (CETSA)

A total of 3 × 10^6^ HCT116 cells were initially seeded. Following a 24 h incubation, the cells underwent treatment with 200 μM UNI66 for 1 h before being harvested. The harvested cells were resuspended in ice-cold PBS containing a protease inhibitor. Subsequently, the samples were subjected to heat treatment at the specified temperatures for 3 min, followed by a 3 min cooling period at room temperature. The samples were then subjected to 2–3 freeze–thaw cycles by immersing the tubes in liquid nitrogen for 5 min and thawing them in a heat block at 25°C for 5 min each. The freeze–thawed cells were centrifuged at 20,000 *g* at 4°C for 20 min. The resulting supernatant was carefully transferred to a new tube, and the protein concentration was quantified using the Bradford assay. The proteins were subsequently analyzed through immunoblotting.

### Zebrafish xenograft

All zebrafish specimens were reared in a circulating aquarium system (Genomic Design, Republic of Korea) maintained at a temperature of 28.5°C, following the guidelines set by the Ulsan National Institute of Science and Technology Institutional Animal Care and Use Committees (IACUC: UNISTIACUC-20-09). To induce vascular visualization in zebrafish embryos, we conducted a mating between the Tg (*fli1:EGFP*) line and WT TAB strain [31]. Fertilized eggs were then cultured in E3 medium until 24 h post-fertilization, after which they were transferred to E3 medium supplemented with 2% phenylthiourea (PTU) for depigmentation purposes. The zebrafish xenograft experiment was performed following previously established protocols [32]. We prepared human colorectal carcinoma HCT116 cells suspended in PBS supplemented with 10% matrigel. For cell labeling, we utilized DiI dye (Vybrant™ DiI Cell-Labeling Solution, Invitrogen V22885). Approximately 1000 labeled cells were then engrafted into 2 day post-fertilization (dpf) zebrafish embryos via injection into the perivitelline space. Microinjection was carried out using a FemtoJet 4i microinjector (Eppendorf) equipped with borosilicate glass needles (Sutter Instrument) fabricated using a PMP-102 Micropipette Puller (MicroData Instrument). For drug treatment, zebrafish embryos from 4 to 6 dpf were exposed to either 7.5 μM of the BET inhibitor JQ1 (Sigma-Aldrich) or 0.075% dimethyl sulfoxide (DMSO) dissolved in E3 medium. Tumor growth was monitored before and after treatment using an AXIO Zoom.V16 fluorescence microscope (Carl Zeiss), and tumor size measurements were conducted using ZEN Blue software (Carl Zeiss). Statistical analyses were performed using Prism 10 software (GraphPad).

### Statistical analysis

The statistical analyses for individual experiments are delineated in the corresponding figure legends. All statistical analyses were conducted using GraphPad Prism (Version 9.0.0). The data are represented as means ± standard error of the mean (SEM) from duplicate or triplicate samples. Significance levels are denoted by *P*-values, where *P*> 0.5 is indicated as not significant (ns), *P*< 0.05 as (*), *P*< 0.01 as (**), *P*< 0.001 as (***), and *P*< 0.0001 as (****).

## Results

### UNI66 exhibited selective cytotoxicity towards PARP1-deficient cells and revertant BRCA2 ovarian cancer cells resistant to PARP inhibitors

**Figure 1.**
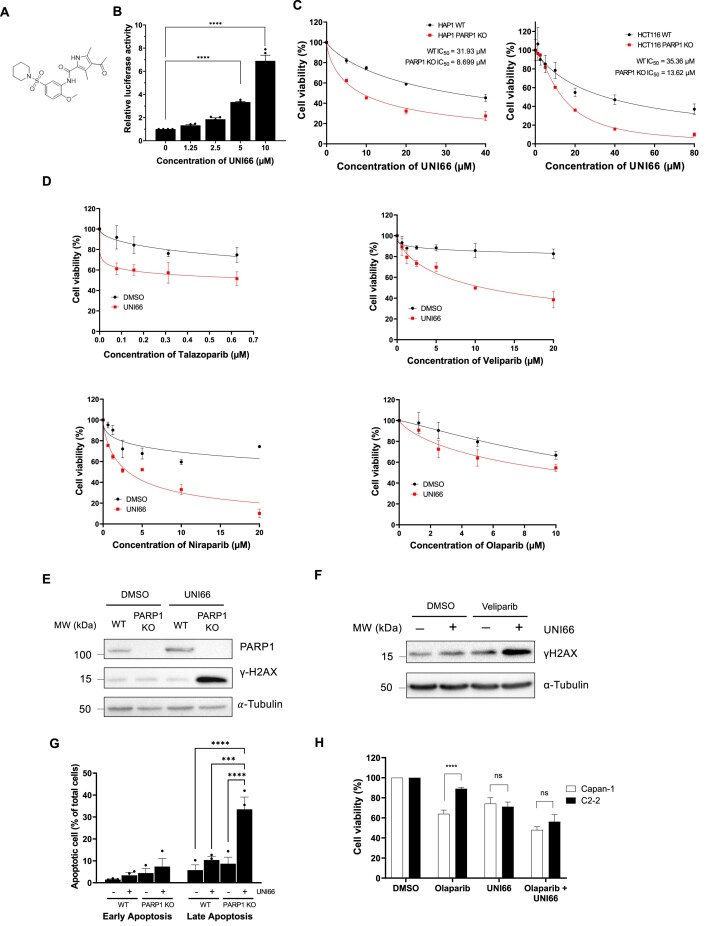
UNI66 selectively kills PARP1-deficient cells. (**A**) Molecular structure of UNI66. (**B**) HEK293T cells, stably expressing ATAD5–luciferase, underwent a 24 h treatment with varying concentrations of UNI66. Luciferase activity was subsequently quantified using One-Glo luciferase reagent. The presented data represent the mean ± SEM from three independent experiments (*n* = 3). Significance was assessed using one-way ANOVA. (**C**) HAP1 and HCT116 cells, cultured in 96-well plates, were exposed to the specified concentrations of UNI66 for 72 h. Cell viability was assessed using Cell Titer-Glo reagent, and the results are displayed as the mean ± SEM (*n* = 3). The IC_50_ was determined through non-linear regression analysis. (**D**) HCT116 cells, cultured in 96-well plates, treated with Talazoparib, Valiparib, Niraparib, or Olaparib at the indicated concentrations in combination with 20 μM UNI66 for 72 h. Cell viability was assessed using Cell Titer-Glo reagent, and data are presented as the mean ± SEM (*n* = 3). (**E**) HCT116 WT and PARP1-deficient cells were exposed to 60 μM UNI66 for 12 h. Following cell harvest, proteins were isolated and subjected to immunoblotting with the specified antibodies. (**F**) HCT116 WT cells were treated with 60 μM UNI66 or 10 μM Veliparib, or with a combination of both. After 12 h, cells were harvested and subjected to immunoblotting. (**G**) HCT116 WT or PARP1-deficient cells underwent treatment with UNI66, and apoptotic cell death was quantified using Annexin V–Alexa Fluor™ 488 conjugate. Flow cytometry analysis was conducted, and data are presented as the mean ± SEM (*n* = 3). Significance was determined using two-way ANOVA. (**H**) Capan and C2-2 cells were treated with 20 μM UNI66 in the absence or presence of 10 μM Olaparib, and their survival was assessed using Cell Titer-Glo reagent. The results are expressed as the mean ± SEM (*n* = 3). Significance was determined using two-way ANOVA.

In our previous studies, we established a high-throughput screening assay utilizing ATAD5 expression as a surrogate indicator for the detection of DNA replication stresses [14]. This assay, applied to screen 344,385 small molecules, identified 289 compounds that enhance DNA replication stresses. Subsequently, 118 of these molecules were selected for further analysis, with 16 demonstrating direct DNA binding, creating impediments to DNA replication, while the remaining 102 exhibited no DNA binding affinity. We postulated that the latter group, lacking DNA binding, might function by inhibiting a crucial enzyme involved in DNA replication and repair. Notably, UNI66, among these molecules, was identified for its ability to elevate ATAD5–luciferase expression without exhibiting DNA binding properties (Fig. 1A, B). In pursuit of identifying synthetic lethal interactions with UNI66, we exposed HAP1 cell lines carrying mutations in *PARP1*, *XPC*,*PRKDC*, *MLH1*,*MUTYH*,*RAD54L*,and *FANCA* to 10 μM UNI66 for 72 h. Remarkably, UNI66 selectively induced cell death in PARP1-deficient cells (Fig. 1C; Supplementary Fig. S1A), while cells with mutations in other genes did not exhibit significant sensitivity differences compared with WT cells (Supplementary Fig. S1B). In addition, UNI66 did not sensitize PARP1-deficient cells further after depletion of PARP2 (Supplementary Fig. S1C). Furthermore, UNI66 exhibited a synergistic cytotoxic effect when combined with PARP inhibitors with varying PARP-trapping abilities, such as Talazoparib, Veliparib, Niraparib, and Olaparib (Fig. 1D).

Given UNI66’s selective lethality towards PARP1-deficient cells, we investigated its impact on the accumulation of DNA damage, as reflected by γ-H2AX levels, in both WT and PARP1-deficient cells post-treatment. In line with the observed differences in cell viability, UNI66-treated PARP1-deficient cells exhibited a dramatic increase of γ-H2AX compared with WT cells (Fig. 1E). Additionally, we observed an increased γ-H2AX level in WT cells when UNI66 is co-treated with Veliparib (Fig. 1F). Additionally, a heightened level of apoptotic cell death was evident in PARP1-deficient cells following treatment with 20 μM UNI66 for 72 h, as compared with WT cells (Fig. 1G). Collectively, UNI66 demonstrates the capacity to selectively induce hypersensitivity, elevate DNA damage levels, and promote apoptotic cell death specifically in PARP1-deficient cells.

One persistent challenge in cancer therapy is the development of resistance to initial drug treatments. Pancreatic cancer cells, such as Capan carrying a BRCA2 mutation and initially sensitive to PARP inhibitors, and the C2-2 clone derived from Capan and exhibiting resistance to PARP inhibitors, have been instrumental in studying PARP inhibitor resistance [33]. Consistent with previous reports, C2-2 cells displayed resistance to the PARP inhibitor Olaparib (Fig. 1H). Intriguingly, although UNI66 treatment exhibited no difference in killing Capan or C2-2 cells, C2-2 cells regained sensitivity to Olaparib upon co-treatment with UNI66. To confirm this result, we utilized TK6 BRCA2-degron cells. UNI66 treatment sensitized BRCA2-degron cells to Talazoparib, even in the absence of auxin for BRCA2 degradation, suggesting that the HR deficiency induced by UNI66 enhances sensitivity to PARP inhibitors (Supplementary Fig. S1D). These observations underscore UNI66’s potential as a strategic intervention, overcoming resistance and restoring sensitivity to PARP inhibitors.

### UNI66 diminishes the efficiency of homologous recombination

**Figure 2.**
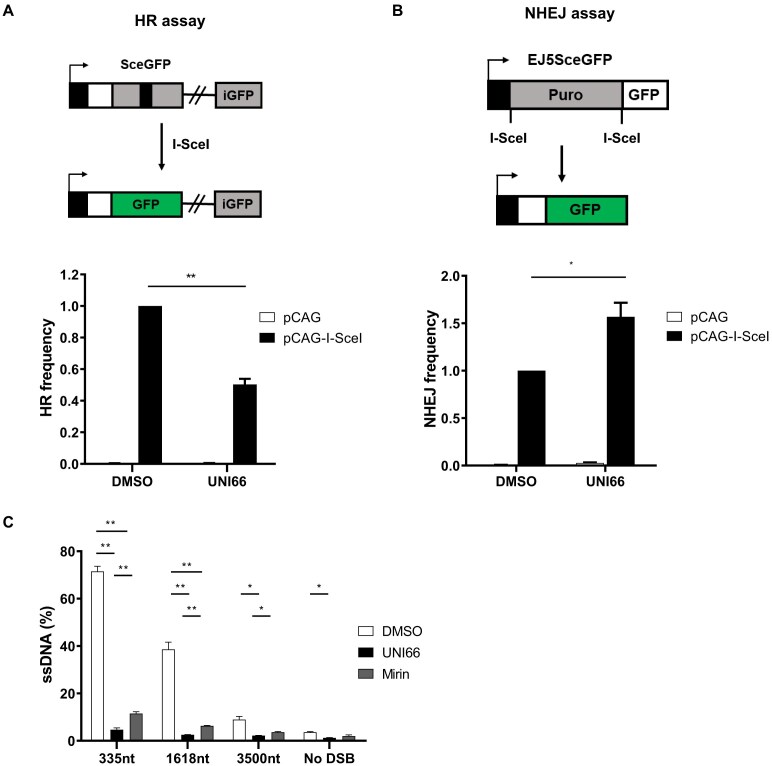
UNI66 reduces HR efficiency. (**A**) HR and (**B**) NHEJ reporter cell lines were transfected with either an empty vector (pCAG) or the I-SceI expression vector (pCAG-I-SceI). Subsequent to a 48 h exposure to 20 μM UNI66, cells were harvested, and the proportion of GFP-positive cells, indicative of HR or NHEJ activity, were quantified by FACS. The data are represented as the mean ± SEM (*n* = 3), and statistical significance was assessed using an unpaired *t*-test. (**C**) U2OS cells, stably expressing ER-AsiSI, underwent treatment with 25 μM Mirin for 48 h or 50 μM UNI66 for 8 h. Genomic DNA was extracted, digested with BsrGI and HindIII enzymes, and the quantification of ssDNA formed by end resection was performed using qPCR. Results are presented as the mean ± SEM (*n*= 3), and significance was determined through an unpaired *t*-test.

The observed selective and synergistic cell killing effect of UNI66 on PARP1-deficient cells prompted an exploration of its impact on HR frequency. We employed the DR–GFP reporter assay, a well-established method for quantifying HR efficiency. This assay utilizes the introduction of a DNA DSB by the I-SceI enzyme between two split GFP cassettes carrying homologous overlapping sequences [34]. HR frequency was diminished by depletion of RAD51, while it remained unaffected by the depletion of LIG4 (Supplementary Fig. S2A). Following UNI66 treatment, we observed a reduction in HR frequency, as indicated by diminished GFP signal production (Fig. 2A). In addition, we measured non-homologous end-joining (NHEJ) efficiency after UNI66 treatment. This system introduces two DNA DSBs between the promoter and GFP cassette, leading to the removal of a long puromycin gene [35]. NHEJ efficiency was decreased by depletion of LIG4, while it remained unaffected by depletion of RAD51 (Supplementary Fig. S2B). We found that UNI66 treatment resulted in an increased efficiency of NHEJ (Fig. 2B). The contrasting effects on HR and NHEJ efficiencies highlight UNI66’s capability to modulate distinct DNA repair pathways. It is possible to use UNI66 as a tool for studying DNA repair mechanisms.

Since POLQ inhibition causes synthetic lethality with BRCA1 and BRCA2 deficiency, we hypothesized that HR reduction by UNI66 would be synthetic lethal with POLQ deficiency [9]. Indeed, we observed that UNI66 mildly sensitized POLQ-deficient cells (Supplementary Fig. S2C).

The initiation of HR involves the crucial step of end resection, where DNA undergoes the generation of long 3′-hydroxyl overhangs. This process is orchestrated by MRE11–RAD50–NBS1 and CtIP for short-range resection, followed by EXO1 or DNA2 for long-range resection [36]. To assess whether UNI66 could impede the efficiency of end resection, we employed the ER-AsiSI assay. This assay enables the measurement of resected ssDNA resistant to the BsrGI restriction enzyme after the introduction of AsiSI-restricted DNA DSBs through PCR amplification [37]. End resection efficiency was decreased by depletion of CtIP, the main factor for early steps of end resection (Supplementary Fig. S2D). Moreover, consistent with previous studies, treatment with the POLQ inhibitor ART558 showed no significant effect on end resection (Supplementary Fig. S2E). We found that UNI66 treatment resulted in a reduction in the efficiency of end resection comparable with the level achieved by Mirin, an inhibitor of MRE11 (Fig. 2C). These findings collectively indicate that UNI66 treatment induces a defect in HR specifically at the end resection step, shedding light on its mechanism of action in modulating DNA repair pathways.

### UNI66 reduces transcription of *CtIP* and *RAD51*

**Figure 3.**
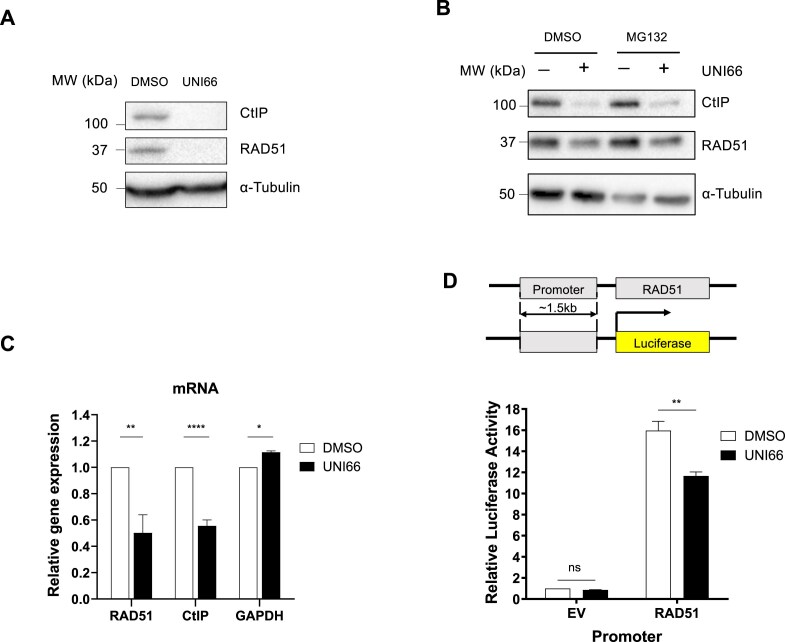
UNI66 reduces transcription of *CtIP* and *RAD51*. (**A**) HCT116 cells were exposed to 60 μM UNI66 for 6 h. The isolated proteins were subjected to immunoblotting using antibodies specific for the indicated proteins. (**B**) HCT116 cells underwent treatment with 50 μM UNI66 for 4 h, followed by a 4 h treatment with 10 μM MG132. The isolated proteins were then subjected to immunoblotting using the specified antibodies. (**C**) Following a 6 h exposure to 60 μM UNI66, HCT116 cells were harvested, and mRNA levels were quantified using RT–qPCR. The data are presented as the mean ± SEM, and statistical significance was determined using an unpaired *t*-test. (**D**) The top panel shows a schematic representation of the *RAD51* promoter–luciferase reporter system. The bottom panel shows results from the *RAD51* promoter–luciferase reporter assay. HCT116 cells were transfected with the RAD51 promoter–luciferase plasmid. After 48 h, the cells were treated with 60 μM UNI66 for 6 h, and luciferase activity was measured using the Dual-Luciferase® Reporter reagent. The data are presented as the mean ± SEM, and significance was assessed using two-way ANOVA.

Given the compromised end resection step of HR by UNI66, we posited that UNI66 might influence the expression of nucleases crucial for HR end resection. Our investigation into the expression of these nucleases upon UNI66 treatment revealed a reduction in the levels of CtIP, EXO1, and MRE11 (Fig. 3A; Supplementary Fig. S3A). Additionally, UNI66 treatment resulted in the down-regulation of RAD51, a protein pivotal for strand invasion during HR (Fig. 3A). Moreover, UNI66 treatment slightly reduced the level of the DNA-dependent protein kinase catalytic subunit (DNA-PKcs) but had no effect on the Ku70 level (Supplementary Fig. S3B). Considering the fluctuating levels of *CtIP* during the cell cycle, the observed decrease in CtIP levels upon UNI66 treatment may be attributed to cell cycle arrest in the G_1_ phase. Since treatment with 60 μM UNI66 for 6 h did not affect the cell cycle (Supplementary Fig. S3C), the reduced CtIP levels do not appear to result from cell cycle changes.

The diminished protein levels of CtIP and RAD51 prompted an exploration into the underlying mechanisms, specifically whether UNI66-induced reductions occur through active proteasomal degradation. To assess this, we measured CtIP and RAD51 protein levels in the presence of 10 μM MG132, a proteasome inhibitor, following treatment with 50 μM UNI66. If UNI66 triggered proteolysis of CtIP and RAD51, MG132 treatment would be expected to restore their protein levels. However, contrary to this expectation, CtIP and RAD51 protein levels remained unrecovered upon inhibition of the proteasomal pathway, suggesting that UNI66 does not reduce CtIP and RAD51 protein levels through proteolysis (Fig. 3B). Subsequently, we investigated whether the diminished protein levels of CtIP and RAD51 have resulted from the inhibition of transcription. As we hypothesized, UNI66 treatment reduced mRNA levels of *CtIP* and *RAD51* (Fig. 3C). To ascertain whether UNI66 influences the promoter activity of the *RAD51* gene, we conducted a luciferase reporter assay. The *RAD51* promoter was placed upstream of the luciferase gene, and luciferase activity was measured following UNI66 treatment. Consistent with the down-regulation of *RAD51* mRNA expression by UNI66, the treatment significantly reduced luciferase expression driven by the *RAD51* promoter (Fig. 3D). Collectively, our findings indicate that UNI66 treatment reduces HR activity by regulating the transcription of the *CtIP* and *RAD51* genes.

### UNI66 binds to BRD4 and inhibits its transcription activation of the *CtIP* and *RAD51* genes

**Figure 4.**
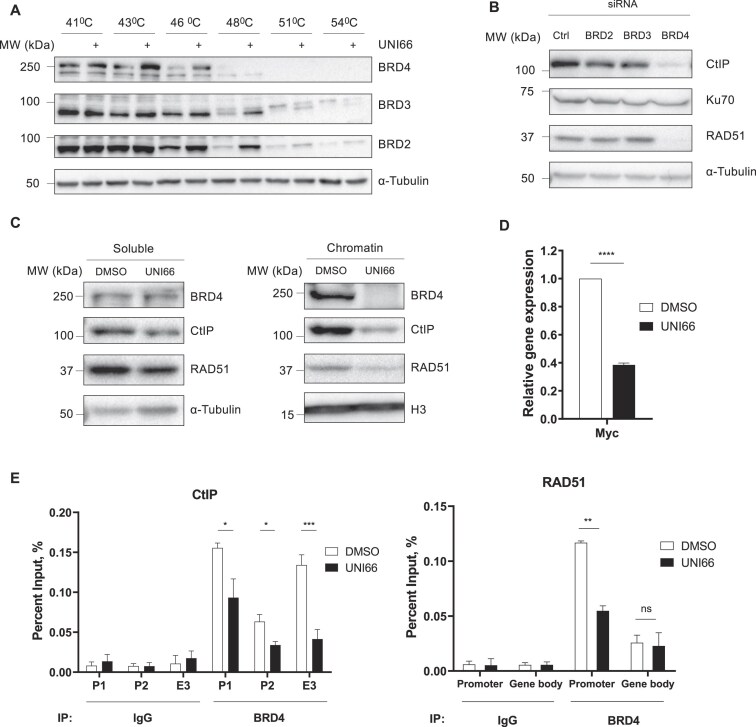
UNI66 binds to BRD4 and reduces the amount of BRD4 on the *CtIP* and *RAD51* promoters. (**A**) HCT116 cells were exposed to 200 μM UNI66 for 1 h and subsequently harvested. After incubation at the indicated temperatures, cells were lysed through freeze–thaw cycles, and the isolated proteins were subjected to immunoblotting using the indicated antibodies. (**B**) HCT116 cells transfected with the indicated siRNAs and incubated for 48 h. The isolated proteins were subjected to immunoblotting using the specified antibodies. (**C**) HCT116 cells, treated with 60 μM UNI66 for 6 h, were harvested, and chromatin fractionation was performed. The isolated proteins were then subjected to immunoblotting using the specified antibodies. (**D**) Following a 6 h treatment with 60 μM UNI66, c-*Myc* mRNA levels were quantified using RT–qPCR. The data are presented as the mean ± SEM, and statistical significance was determined through an unpaired *t*-test. (**E**) After a 6 h exposure to 60 μM UNI66, HCT116 cells were subjected to ChIP to assess BRD4 accumulation at corresponding promoter and enhancer regions. The data are presented as the mean ± SEM, and significance was determined through an unpaired *t*-test (P, promoter; E, enhancer).

To identify the target protein of UNI66, we conducted an exploration of the PubChem database within the NIH Molecular Library Probe Production Centers Network (MLPCN) library (https://commonfund.nih.gov/molecularlibraries/index). Notably, our investigation revealed that small molecules structurally similar to UNI66 were categorized as potential inhibitors of BET family proteins. We assessed the binding affinity of UNI66 for BET family proteins using the CETSA. This assay measures the interaction between a ligand and its binding protein by monitoring the thermal stabilization of the target protein upon ligand interaction [38]. Following a 1 h incubation with UNI66, we observed an increase in the thermal stability of BRD2, BRD3, and BRD4 at high temperatures (Fig. 4A), suggesting an interaction between UNI66 and these BET family proteins. Since UNI66 treatment reduces CtIP and RAD51 levels, we examined which BET proteins regulate their expression. Only BRD4 depletion, not that of BRD2 or BRD3, decreased CtIP and RAD51 levels, indicating BRD4 as their regulator (Fig. 4B; Supplementary Fig. S4A).

BET family proteins recognize and bind to acetylated lysine residues on histones for transcriptional activation of target genes, we hypothesized that UNI66 might disrupt BRD4 binding to chromatin, leading to a reduction in CtIP and RAD51 transcription. As we hypothesized, UNI66 inhibited the binding of BRD4 to chromatin (Fig. 4C). In addition, UNI66 treatment significantly reduced the transcription of c-*Myc* (Fig. 4D), a well-known BRD4 target gene, providing further evidence that UNI66 inhibits BRD4 function.

To gain deeper insight into the mechanism by which UNI66 inhibits BRD4, we employed the well-known BRD4 inhibitor JQ1. This comparative approach allows us to elucidate the functional dynamics of UNI66 in relation to JQ1, helping to clarify any unique or overlapping pathways affected by both compounds. Intriguingly, similar to UNI66, JQ1 treatment increased NHEJ activity (Supplementary Fig. S4B). To check whether the compounds have an additional effect, we assessed HR activity following co-treatment with UNI66 and JQ1. We found that HR activity was down-regulated by treatment with each compound, but was not affected further by co-treatments (Supplementary Fig. S4C). In addition, JQ1 and UNI66 treatments do not have an additional effect in killing PARP1 knockout (KO) cells, which is consistent with the HR data (Supplementary Fig. S4D), suggesting that UNI66 and JQ1 may have a similar mechanism of action in their inhibition of BRD4.

BRD4 inhibitors function by competing with acetylated residues for binding at the BRD4 bromodomains, thereby displacing BRD4 from super enhancers and promoters [39]. To investigate whether UNI66 has a similar mechanism of action, we examined the presence of BRD4 on the promoters of *CtIP* and *RAD51* by ChIP. UNI66 treatment reduced the accumulation of BRD4 protein on the c-*Myc* promoter, but not on the *WRN* promoter (Supplementary Fig. S4E). Moreover, UNI66 treatment led to a reduction in the presence of BRD4 on the promoters of *CtIP* and *RAD51* (Fig. 4E). These findings collectively support the conclusion that UNI66 inhibits BRD4 from binding to the promoters of its target genes, including *CtIP* and *RAD51*.

### BRD4 inhibition effectively mitigated the growth of tumors lacking PARP1 activity

**Figure 5.**
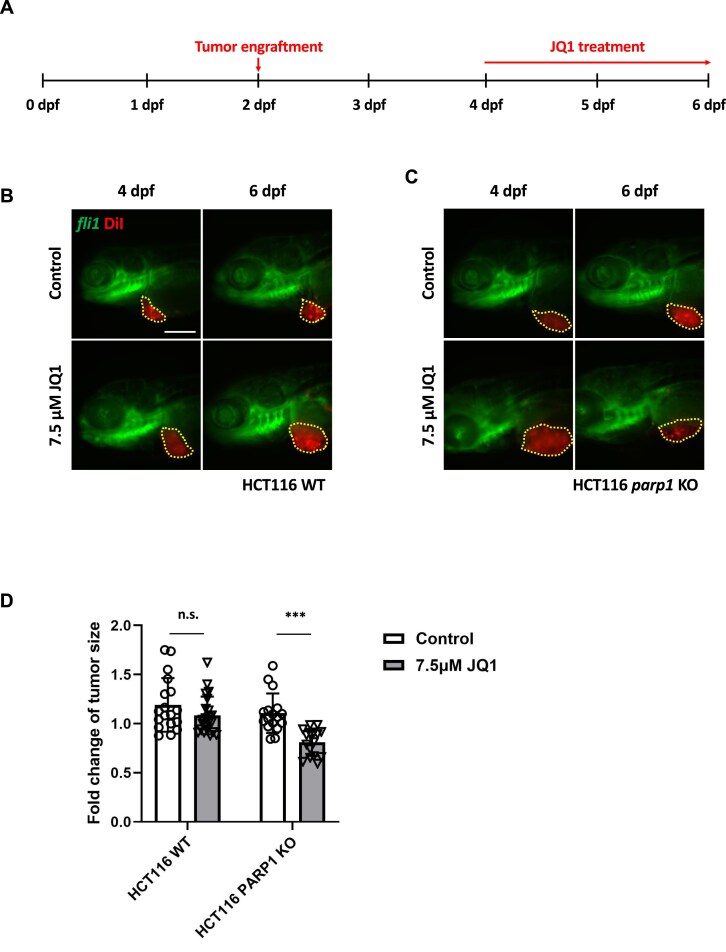
Inhibition of BRD4 attenuates tumor growth in a zebrafish xenograft model. (**A**) Schematic depiction outlining the procedure for the xenograft and BRD4 inhibition experiment in zebrafish. (**B**) Representative fluorescence microscopy image illustrating HCT116 WT tumor-engrafted embryos with or without JQ1 treatment. (**C**) Representative fluorescence microscopy image demonstrating HCT116 *parp1* KO tumor-engrafted embryos with or without JQ1 treatment. (**D**) Quantitative assessment of tumor size alteration following control (DMSO) or JQ1 treatment. The graph depicts the mean ± SEM with individual data points. Statistical significance was determined using an unpaired two-tailed Student’s *t*-test. *P < 0.05; n.s., not significantly different. Scale bar = 200 μm. The yellow-dotted region in (B) and (C) delineates DiI-positive tumors.

To ascertain whether BRD4 inhibition, resulting in transcriptional suppression of *CtIP* and *RAD51*, specifically curtailed tumor growth in the absence of PARP1 *in vivo*, we xenografted colorectal carcinoma cells, including HCT116 and isogenic *parp1* KO cells, into fertilized zebrafish embryos. Since UNI66 demonstrated no tumor growth inhibition, potentially attributable to solubility challenges in achieving effective treatment concentrations for zebrafish xenograft experiments (Supplementary Fig. S5A), we employed JQ1, a widely recognized BRD4 inhibitor, for an *in vivo* experiment aimed at tumor suppression. As expected, JQ1 exhibited a heightened lethality towards PARP1-deficient HCT116 cells *in vitro* (Supplementary Fig. S5B). Upon tumor establishment in hatched zebrafish embryos, treatment with 7.5 μM BRD4 inhibitor JQ1 was administered. While WT HCT116 cells exhibited no discernible reduction in tumor growth upon JQ1 treatment, the growth of *parp1* KO HCT116 tumors was significantly inhibited (Fig. 5). These findings collectively underscore the transcriptional down-regulation of HR genes, notably *CtIP* and *RAD51*, by BRD4 inhibition as a mechanism for suppressing PARP1-deficient tumor growth.

## Discussion

PARP inhibitors exploit synthetic lethality to target HR-deficient cancer cells. In the classical model, HR-deficient cells fail to repair DSBs caused by SSB repair defects or PARP trapping [5, 40]. Alternative models suggest that ssDNA gaps are also lethal in these cells. PARP inhibitors exacerbate ssDNA gap accumulation [7] or prolong their persistence [41], making BRCA1/2-deficient cells unable to manage them effectively [42]. The success of PARP inhibitors in cancer treatment has been remarkable, but the emergence of resistance to these inhibitors presents significant challenges for patients undergoing such therapy. A key mechanism contributing to PARP inhibitor resistance is the restoration of HR. Consequently, the development of drugs targeting HR or gaining insights into the mechanisms controlling the expression of HR genes holds great promise for treating tumors that have acquired resistance to PARP inhibitors [33]. Thus, understanding the intricate mechanisms governing HR provides significant advantages in devising treatments for HR-proficient or PARP inhibitor-resistant tumors. HR regulation occurs at multiple stages, offering a comprehensive approach to intervention. Transcriptional regulation represents one facet of HR modulation. Transcription factors such as p53 [43] and E2F1 [44] can positively regulate HR genes, including RAD51, by influencing their transcription. Chromatin modifiers, exemplified by CHD4, play a crucial role in RAD51 transcription by binding to the RAD51 promoter and enhancing the acetylation of H3K9 [45]. Beyond transcriptional control, HR gene expression undergoes post-transcriptional regulation. Long non-coding RNA, such as Lnc-RI, has been identified as a stabilizer of *RAD51* mRNA [46]. The transport of HR gene mRNA, such as of *RAD51*, from the nucleus to the cytoplasm also contributes to the modulation of HR gene expression [47]. Furthermore, the protein levels of HR components can be regulated through mechanisms affecting protein stability. Recent findings from our research indicate that specific ubiquitin ligases can selectively regulate HR proteins, including RAD51, CtIP, and CHK1. These intricate layers of regulation underscore the complexity of HR control, offering multiple points of intervention for therapeutic strategies. Previous studies have indicated that BRD4 can be a valuable component of combination therapy with PARP inhibitors. Inhibition of BRD4 has been shown to re-sensitize PARP inhibitor-resistant cells to Olaparib [25]. Several BRD4 inhibitors have been developed, including JQ1. BRD4 inhibitors function by competing with acetylated residues for binding at the BRD4 bromodomains, thereby displacing BRD4 from super enhancers and promoters [39]. Consistent with these findings, UNI66 interacts with BRD4 and inhibits its localization to the promoters of *CtIP* and *RAD51* similar to the mechanism of the other BRD4 inhibitors. However, due to *in vivo* solubility issues and possible off-target effects, it is necessary to develop more potent derivatives for clinical use.

Genetic and epigenetic alterations contribute to disruptions in transcriptional processes, creating a scenario where cancer cells become heavily dependent on specific regulators of gene expression [48]. Changes in transcription factors or chromatin regulators have been identified as influential factors in shaping a tumor’s response to DNA damage, leading to enhanced survival and resistance to treatment [49]. BRD4, functioning as a chromatin reader, plays a pivotal role in activating the transcription of its target genes. Prior studies have indicated that inhibition of BRD4 results in a reduction in HR activity [25, 26]. Our findings, demonstrating that UNI66 binds to BRD4 and disrupts its localization to the promoters and enhancers of *CtIP* and *RAD51*, strongly support the proposition that inhibiting BRD4 could be a promising therapeutic option for the treatment of PARP inhibitor-resistant tumors. By interfering with BRD4, UNI66 presents a mechanism to modulate HR activity, potentially overcoming resistance mechanisms and sensitizing cancer cells to treatment. This insight into the regulatory role of BRD4 further underscores the potential of targeting chromatin regulators in precision cancer therapy. However, due to the pleiotropic effects of BRD4 on the transcription of multiple genes, other mechanisms influencing synthetic sensitization with PARP inhibition cannot be completely excluded. Given BRD4’s role as a key gene regulator, understanding transcriptional changes following its inhibition is crucial. We found that JQ1 and UNI66 treatments reduced EXO1 and MRE11 expression, warranting further investigation into BRD4’s effects on these genes. While reduced NHEJ activity is known to restore HR [50, 51], how reduced HR enhances NHEJ remains unclear. Notably, both treatments increased NHEJ activity, probably due to BRD4 inhibition, highlighting the need for further mechanistic studies in future.

This study unveils UNI66 as a novel inhibitor of BRD4, presenting a unique mechanism by which it regulates HR. UNI66 exerts its influence on HR by impeding the localization of BRD4 to the promoters of *CtIP* and *RAD51*, resulting in the suppression of their transcription. Notably, UNI66 as well as JQ1, a well-known BRD4 inhibitor, exhibits selective lethality towards PARP1-deficient cells, suggesting a potential sensitizing effect of BRD4 inhibition for the PARP inhibitor. Our findings shed light on a subset of HR genes regulated at the transcriptional level, emphasizing the significance of targeting the transcriptional control mechanisms of HR genes.

## Supplementary Material

zcaf013_Supplemental_File

## Data Availability

The data underlying this article are available in the article and in its online supplementary material.

## Notes

present address: asan institute for life sciences, asan medical center, seoul 05505, republic of korea
