# Supplementary material for: Synergistic enhancement of PARP inhibition via small molecule UNI66-mediated suppression of BRD4-dependent transcription of RAD51 and CtIP
Source: NAR Cancer. 2025 Apr 30;7(2):zcaf013. doi: 10.1093/narcan/zcaf013 (PMC12041917; doi:10.1093/narcan/zcaf013)
Supplement: zcaf013_Supplemental_File [file zcaf013_supplemental_file.pdf]

Synergistic Enhancement of PARP Inhibition via Small Molecule UNI66-Mediated Suppression of BRD4-Dependent Transcription of *RAD51* and *Ctip*

Supplementary Information

By

Enkhzul Amarsanaa[1,2]*, Minwoo Wie[1,2,5]*, Unbeom Shin[1], Nabeela Bilal[1,3], Jungme Hwang[1], Eun A Lee[1], Seon Young Lee[1], Byung-Gyu Kim[1], Shinseog Kim[1], Yoonsung Lee[4], and Kyungjae Myung[1,3+]

1. Center for Genomic Integrity, Institute for Basic Science, Ulsan 44919, Republic of Korea.

2. Department of Biological Sciences, Ulsan National Institute of Science and Technology, Ulsan 44919, Republic of Korea

3. Department of Biomedical Engineering, Ulsan National Institute of Science and Technology, Ulsan 44919, Republic of Korea

4. Clinical Research Institute, Kyung Hee University Hospital at Gangdong, School of Medicine, Kyung Hee University, Seoul 05278, Republic of Korea

5. Current address: Asan Institute for Life Sciences, Asan Medical Center, Seoul, 05505, Republic of Korea

*These authors equally contributed.

[+]Correspondence to Kyungjae Myung: E-mail kmyung@ibs.re.kr; Phone +82-52-217-5323; Fax +82-52-217-5519

Supplementary Figure Legends

Supplementary figure 1. UNI66 selectively kills PARP1-deficient cells.

A. HCT116 WT and PARP1-deficient cells were treated with UNI66 for 10 days. Cells were stained with methylene blue to determine clonogenic survival. Data are presented as mean ± SEM (n=3). **B.** HAP1 WT and DNA repair deficient cells were treated with 10 µM UNI66 for 72 hours. Cell viability was measured by CellTiter-Blue reagent. **C.** Left panel: HCT116 WT or PARP1-deficient cells were transfected with control and PARP2 siRNAs. After 2 days, cells were seeded in 96 well plates and treated with indicated concentrations of UNI66 for 72 hours. Cell viability was measured by Cell Titer-Glo reagent. Right panel: HCT116 cells were transfected with control and PARP2 siRNAs. After 2 days, cells were harvested, and mRNA levels were analyzed by using RT-qPCR. The data are presented as mean ± SEM, and significance was determined using an unpaired t-test. **D.** TK6 BRCA2-degron cells were treated with 200 µM Auxin. The following day, cells were seeded in 96 well plates, treated with 0.15 µM Talazoparib and 20 µM UNI66 and incubated for 72 hours. Cell viability was measured by CellTiter-Blue reagent. The data are presented as mean ± SEM, and significance was determined using two-way ANOVA.

Supplementary figure 2. UNI66 reduces homologous recombination efficiency.

A. HR and **B.** NHEJ Reporter Cell Lines were transfected with RAD51 and LIG4 siRNAs for control. The following day, cells were subsequently transfected with either an empty vector or I-*Sce*I expression vectors. After 48 hours, GFP-positive cells were measured by FACS. The data are represented as mean ± SEM (n=3), and statistical significance was assessed using an unpaired t-test. C, D. **C.** U2OS WT and POLQ KO cells were seeded in 96 well, treated with indicated concentrations of UNI66 and incubated for 72 hours. Cell viability was measured by CellTiter-Blue reagent. The data are presented as mean ± SEM (n=3). **D, E.** DNA end resection was measured using U2OS cells stably expressing ER-*AsiS*I after transfection of either control or CtIP siRNAs **(D)** or treatment with 10 µM ART558 **(E)**. After 48 hours, genomic DNA was extracted, digested with B*srG*I and H*ind*III

enzymes, and the quantification of single-strand DNA formed by end resection was performed using qPCR. Results are presented as mean ± SEM (n=3), and significance was determined through an unpaired t-test.

Supplementary figure 3. UNI66 reduces expression of CtIP and RAD51

A, B. HCT116 cells were subjected to a 6-hour treatment with 60 μM UNI66, and the isolated proteins were subsequently immunoblotted using the indicated antibodies. **C.** HCT116 cells were treated with 60 μM UNI66 for 6 hours. The cell cycle profiles were analyzed by FACS.

Supplementary figure 4. UNI66 binds to BRD4 and inhibits its transcription activation of the CtIP and RAD51 genes.

A. HCT116 cells were transfected with indicated siRNAs. After 48 hours, isolated proteins were analyzed using immunoblotting. **B.** NHEJ reporter cell lines were transfected with either an empty vector or I-*Sce*I expression vector. The following day, cells were treated with indicated concentrations of JQ1 for 24 hours. The proportion of GFP-positive cells were quantified by FACS. The data are represented as mean ± SEM (n=3), and significance was assessed using an unpaired t-test. **C.** HR reporter cell lines were transfected with either an empty vector or I-*Sce*I expression vector. The following day, cells were treated with JQ1 or combined treatment with UNI66 at indicated concentrations. The proportion of GFP-positive cells were quantified by FACS. The data are represented as mean ± SEM (n=3), and statistical significance was assessed using one-way ANOVA. **D.** HCT116 WT and PARP1 KO cells were seeded in 96 well plate. The following day, cells were treated with indicated concentrations of UNI66 and 0.5 μM JQ1 and incubated for 72 hours. Cell viability was measured by CellTiter-Blue reagent. The data are represented as mean ± SEM (n=3). **E.** HCT116 cells were treated with 60 μM UNI66 for 6 hours and subjected to chromatin immunoprecipitation to assess BRD4 accumulations at Myc and WRN promoters. The data are represented as mean ± SEM (n=3), and significance was assessed using an unpaired t-test.

Supplementary figure 5: Treatment with the BRD4 inhibitor, JQ1, reduces the expression of HR genes and sensitizes PARP1 knockout cells similar to UNI66.

A. Quantitative assessment of tumor size alternation following different doses of UNI66. The graph depicts mean ± standard error of the mean (S.E.M.) with individual data points. **B.** HCT116 wild-type and PARP1 knockout cells were treated with indicated doses of JQ1 for 48 hours. Cell survival was assessed using the Cell Titer-Glo reagent. The results are expressed as mean ± SEM (n=3).

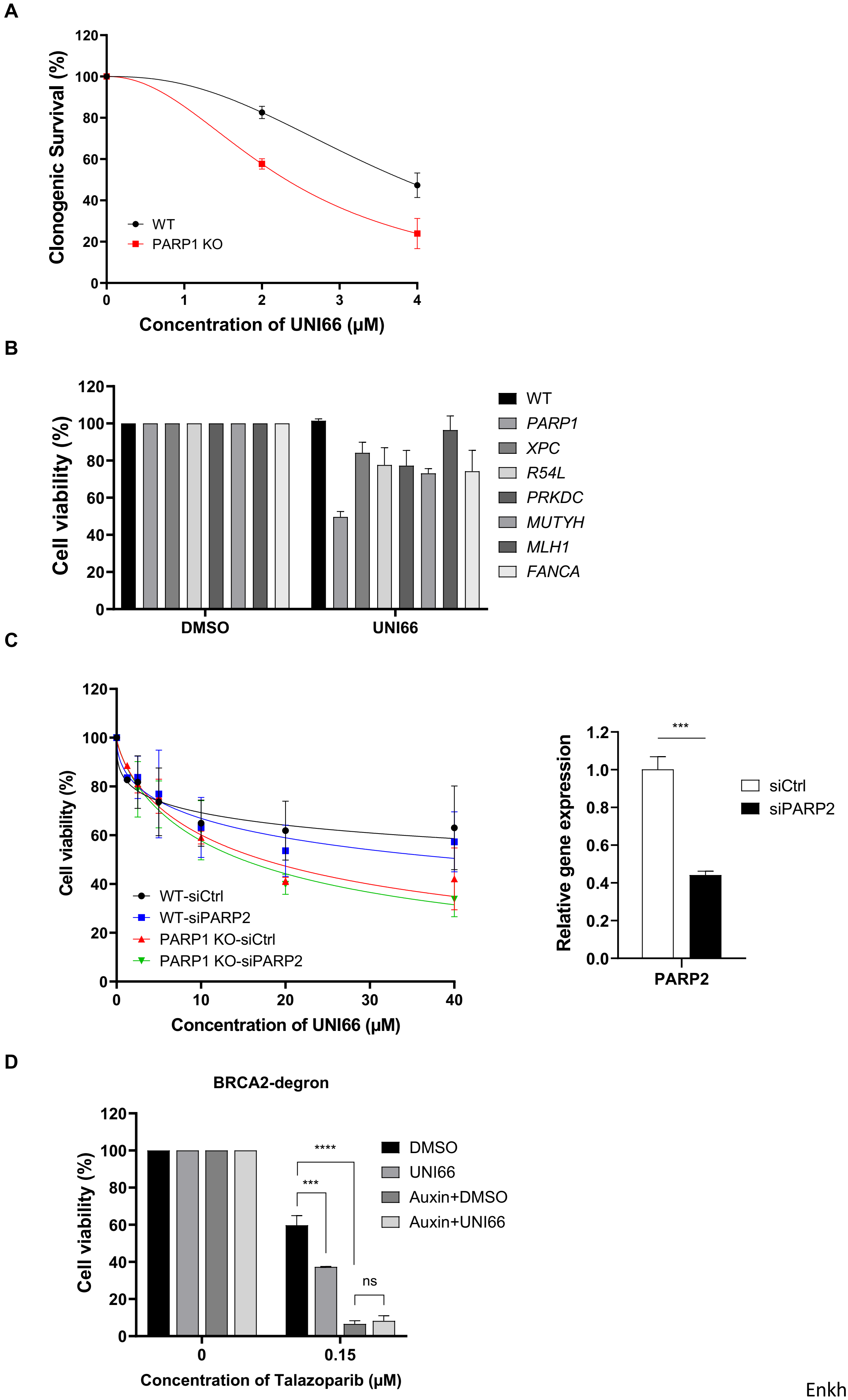

Enkhzul et al Fig S1

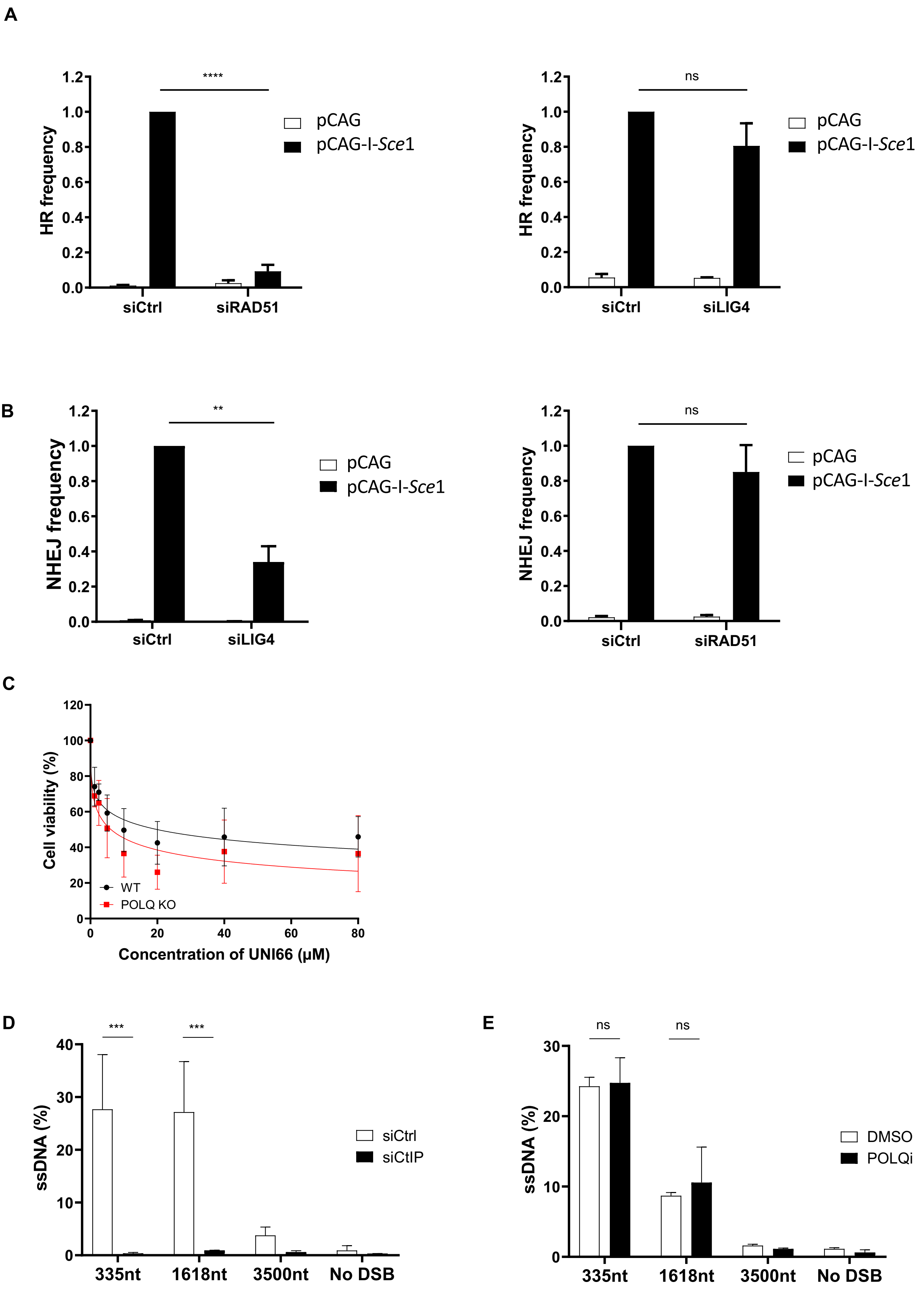

Enkhzul et al Fig S2

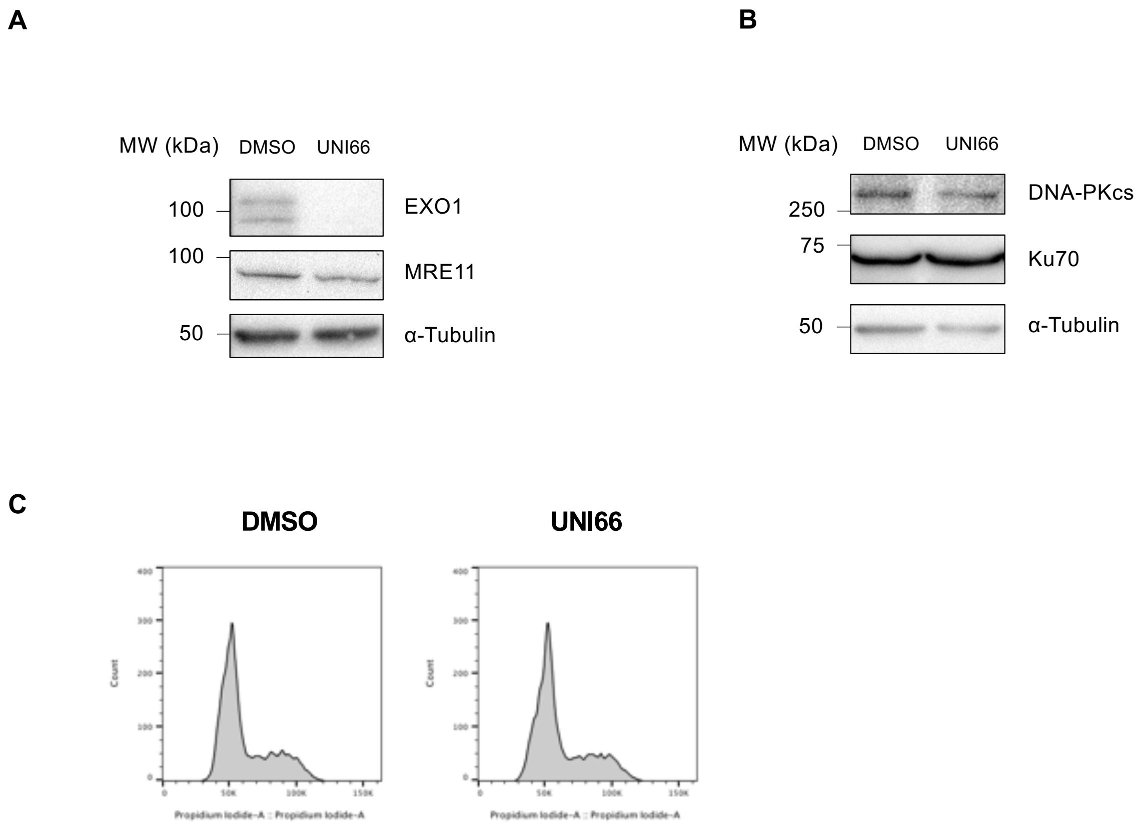

Enkhzul et al Fig S3

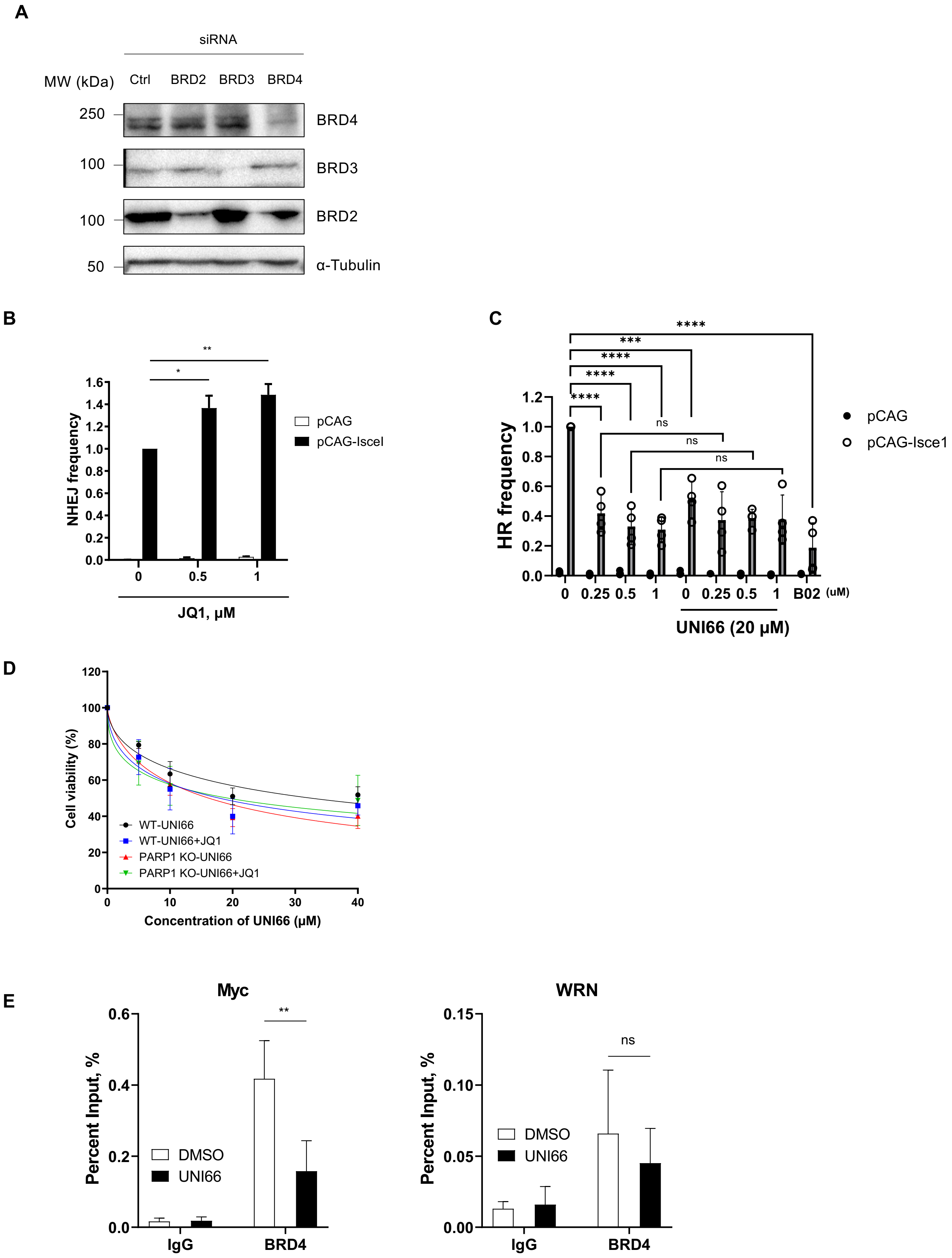

Enkhzul et al Fig S4

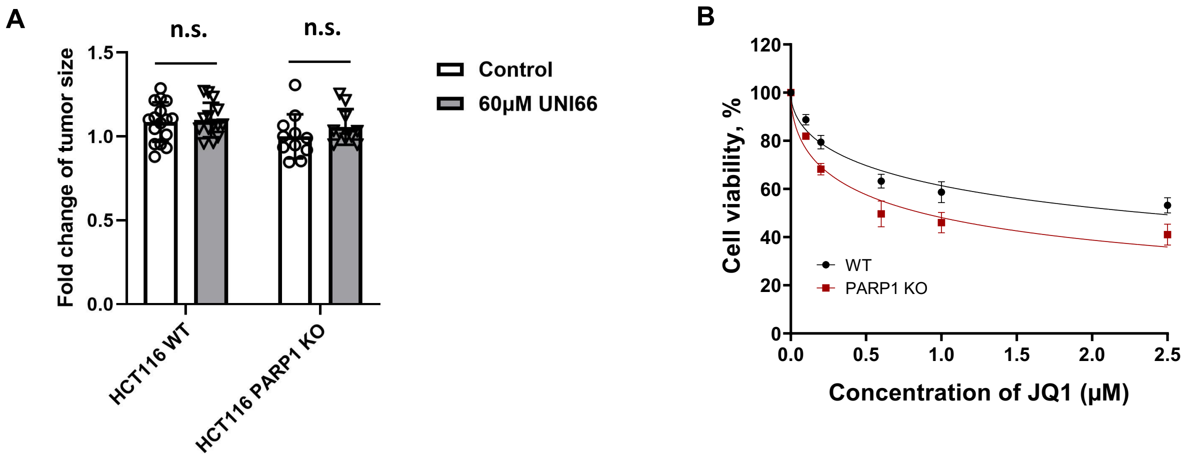

Enkhzul et al Fig S5
